# Productivity performance of peach trees, insecticidal and antibacterial bioactivities of leaf extracts as affected by nanofertilizers foliar application

**DOI:** 10.1038/s41598-021-89885-y

**Published:** 2021-05-13

**Authors:** Walid F. A. Mosa, Ahmed M. El-Shehawi, Marwa I. Mackled, Mohamed Z. M. Salem, Rehab Y. Ghareeb, Elsayed E. Hafez, Said I. Behiry, Nader R. Abdelsalam

**Affiliations:** 1 Plant Production Department (Horticulture-Pomology), Faculty of Agriculture, Saba Basha, Alexandria University, Alexandria, 21531 Egypt; 2 Department of Biotechnology, College of Science, Taif University, P.O. Box 11099, Taif, 21944 Saudi Arabia; 3 Department of Stored Product Pests, Plant Protection Institute, Agriculture Research Center (ARC), Sabahia, Alexandria Egypt; 4 Forestry and Wood Technology Department, Faculty of Agriculture (EL-Shatby), Alexandria University, Alexandria, Egypt; 5 Plant Protection and Biomolecular Diagnosis Department, Arid Lands Cultivation Research Institute, City of Scientific Research and Technological Applications, New Borg El-Arab, Alexandria, 21934 Egypt; 6 Agricultural Botany Department, Faculty of Agriculture, Saba Basha, Alexandria University, Alexandria, 21531 Egypt

**Keywords:** Genetics, Plant sciences, Biological techniques, Biological models, Cytological techniques, Genetic techniques, Mass spectrometry

## Abstract

The current study was performed on eight years old peach (*Prunus persica* L. Batsch) trees cv. Florida prince to study the influence of spraying of commercial nano fertilizer on vegetative growth, pollen grain viability, yield, and fruit quality of the "Florida prince" peach cultivar. Furthermore, extracts from the nanofertilizer treated leaves were studied for their bioactivity as insecticidal or bactericidal activities against some stored grain insects and plant bacterial pathogens. Seventy uniform peach trees were sprayed three time as follow: before flowering; during full bloom, and one month later in addition using the water as a control. Commercial silver particales (Ag NPs) at 10, 12.5, and 15 mL/L and zinc particales (Zn NPs) at 2.5, 5 and 7.5 mL/L as recommended level in a randomized complete block design in ten replicates/trees. Spraying Ag NP at 15 mL/L increased shoot diameter, leaf area, total chlorophyll, flower percentage, fruit yield and fruit physical and chemical characteristics, followed by Ag NPs at 12.5 mL/L and Zn NPs at 7.5 mL/L. Moreover, Zn and Ag NPs caused a highly significant effect on pollen viability. Different type of pollen aberrations were detected by Zn NPs treatment. The commercial Ag NPs showed a high increase in pollen viability without any aberrations. The Ag NPs significantly increased the pollen size, and the spores also increased and separated in different localities, searching about the egg for pollination and fertilization. Peach leaves extract was examined for their insecticidal activity against rice weevil (*Sitophilus oryzea* L.) and the lesser grain borer (*Rhyzopertha dominica,* Fabricius) by fumigation method. The antibacterial activity of all treatments was also performed against molecularly identified bacteria. Ag NPs treated leaves extract at concentration 3000 µg/mL were moderate sufficient to inhibit all the bacterial isolates with inhibition zone (IZ) ranged 6–8.67 mm with high efficiency of acetone extracts from leaves treated with Ag NPs compared with Zn NPs*.* Also, *S. oryzae* was more susceptible to acetone extracts from leaves treated with both nanomaterials than *R. dominica.*

## Introduction

Peach (*Prunus persica* L. Batsch.) belongs to the family of Rosacea is considered as one of the nutritionally and economically important fruits, popular fruits consumed worldwide, and the cultivated area in Egypt is 15,748 hectare which produced 358,012 Mg^1^. Several studies reported the importance of nanofertilizers that could be used in small quantities rather than widespread fertilizes^2–8^. The application of NPs stimulated the plant growth and crop yield and reduced chemical fertilizers usage, so it takes a lot of interest^8–11^. Moreover, DeRosa et al.^12^ reported that nanofertilizers are beneficial in inhibiting the losing of nutrients from the soil, so they help in reducing the soil pollution by avoiding the excessive mineral fertilizers^7,13,14^. Besides, these nanofertilizers can avoid the interaction between nutrients, air, water, microorganisms, and soil. The foliar application of nanofertilizers provides nutrients with high efficiency and low waste due to their faster and higher translocation to different parts of plants^15,16^. Nanoparticles are characterized by small size, low weight, and a high surface to volume ratio^8,17–20^.

Ag NPs has a great impact on growth and advancement of plants such as germination, the ratio of root- shoot, growing of seeds, root growing and elongation and inhibiting of senescence^21,22^. Also, Sharma et al.^23^ mentioned that Ag NPs are distinguished by their unparalleled physiochemical and biological properties comparing with macro-scaled counterparts of it. Besides, the addition of Ag NPs at 20–60 ppm stimulated the plant growth, leaf area, shoot and root length and seed content from chlorophyll, carbohydrate, protein and enzymes of antioxidants in common bean, and corn^24^ and in mustard greens^25^. Ag NPs exhibited strong biological activity^26^ where they influence plants at many different levels^6,27,28^. Besides^29^, stated that 25 ppm Ag NPs improved significantly leaf area and grain yield while, the 75 ppm treatment decreased the grain yield yield. Ag NPs affected plant growth invigoration^25,29,30^, enhanced pigment content^31^, increased biomass accumulation^32^, improved shoot induction and proliferation^33^. It was noticed by many authors that the lack of Zn minimized the level of chlorophyll and photosynthetic rate in plants^34–36^. Zn plays an important role in enhancing the photosynthesis and fruit number per plant and minimizing the abscission of flowers and fruits^37–39^. ZnO NP's application had a positive effect on seed germination, seedling vigor, leaf chlorophyll content, and growth of stem and root in peanut^40^. Moreover^41^, found that spraying Zn NPs chelate fertiliser at 120 mg Zn/L on pomegranate (*Punica granatum* cv. Ardestani) increased fruit yield, by increasing the fruit number per tree and increased TSS, and decreased TA. Zinc plays an essential role in plant functions. It modifies auxin effects through the regulation of tryptophan synthesis^42^.

Soft rot or black-leg bacteria are dangerous microbes that, in past years, could damage many crop plants. Many authors have established methods to diagnose or classify the soft rot pathogen and provide control methods^43,44^. *Dickeya* spp is aggressive to the potato plants, have been isolated from infected plants in several European and Middle East countries which found to be very, especially in Egypt and Israel than *Pectobacterium atrosepticum* bacteria^45^. *Serratia pylumthica* is an ubiquitous bacterium recovered from the rhizosphere worldwide, both as a free-living and endophytic organism bacteria and associated with many plant pathogens^46,47^.

One of the greatest destructive apple and pear bacterial diseases is fire blight, caused by the necrogenic Gram-negative bacterium *Erwinia amylovora*. Since there are no effective control measures available, this disease poses an significant threat to pome production^45,48^. In order to distinguish the phylogenetically closely related species caused by *Agrobacterium tumefaciens* and other species, 16S rDNA gene was developed as a rapid detection means for analyzing and distinguishing strains that belonged to all microbial species^49^.

The insect or bacteria resistance against synthetic pesticides considers an important reason to find other natural plant materials to decrease resistance and environmental pollution. Many previous studies reported that natural plant extract and their essential oils could be used as alternatives for synthetic insecticides or bactericides^45,50–54^. The plant isolates can also affect insect behavior, for instance, prohibiting feeding activity, pest physiology, respiratory inhibition, growth and fecundity reduction, and cuticle disruption^55–57^. Plant extracts are environmentally attractive molecules because it is biodegradable and have negligible side effects on non-target organisms^58–60^. Numerous plant extracts that contain substances with insecticidal effects also include a large group of the so-called essential oils. Numerous studies have termed the toxicity of EOs and plant extracts, such as fumigants, repellent, larvicide, insecticide, and insect growth regulator as well as their compound, against several stored product insects^53,61,62^.

Pollen viability includes several aspects of pollen performance like fertilization ability, germinability, and stainability^52,63–65^. Conventional techniques to test the pollen viability are staining techniques, in vitro germination, seed set and in vivo, and semi-in situ germination on the excised stigma, also named stigmatic germination^66–68^. The **s**taining techniques of pollen grains aims to imagine specific compounds, contents, or cellular compartments related to pollen viability based on different type such as potassium iodide, acetocarmine stain starch, aniline blue and, callose, and chromatin, and the absence of colors indicate non-viable pollen^69,70^. Although the staining techniques present the opportunity to distinguish aborted and non-aborted fresh pollen, they regularly fail to discriminate various viability levels^71^.

Leaves and stems can be a viable source for natural phenolic compounds. From leaves and stems as by-products from peach, several phenolic and flavonoid compounds (chlorogenic acids, catechin, epicatechin, syringic, ferulic, coumaric acids, and quercetin-3-galactoside) and volatile substances benzaldehyde, myrcene, and terpinolene were identified with high antioxidant activities^72^. Chlorogenic acid, catechin, epicatechin, rutin, and cyanidin‐3‐glucoside were detected as ripened peach fruits' main phenolic compounds^73,74^. Polyphenolic compounds like procyanidin, cyanidin, phloridzin, naringenin, and chlorogenic acid were isolated from the peach pulp^18,75^.

Therefore, this investigation aims to study the influence of spraying of commercial nano fertilizer on vegetative growth, pollen grain viability, yield, and fruit quality of the "Florida prince" peach cultivar. Furthermore, extracts from the nano fertilizer treated leaves were studied for their bioactivity as insecticidal or bactericidal activities against some stored grain insects and plant bacterial pathogens. Phenolic compounds from leaf extracts were determined with HPLC.

## Materials and methods

**Scheme 1:**
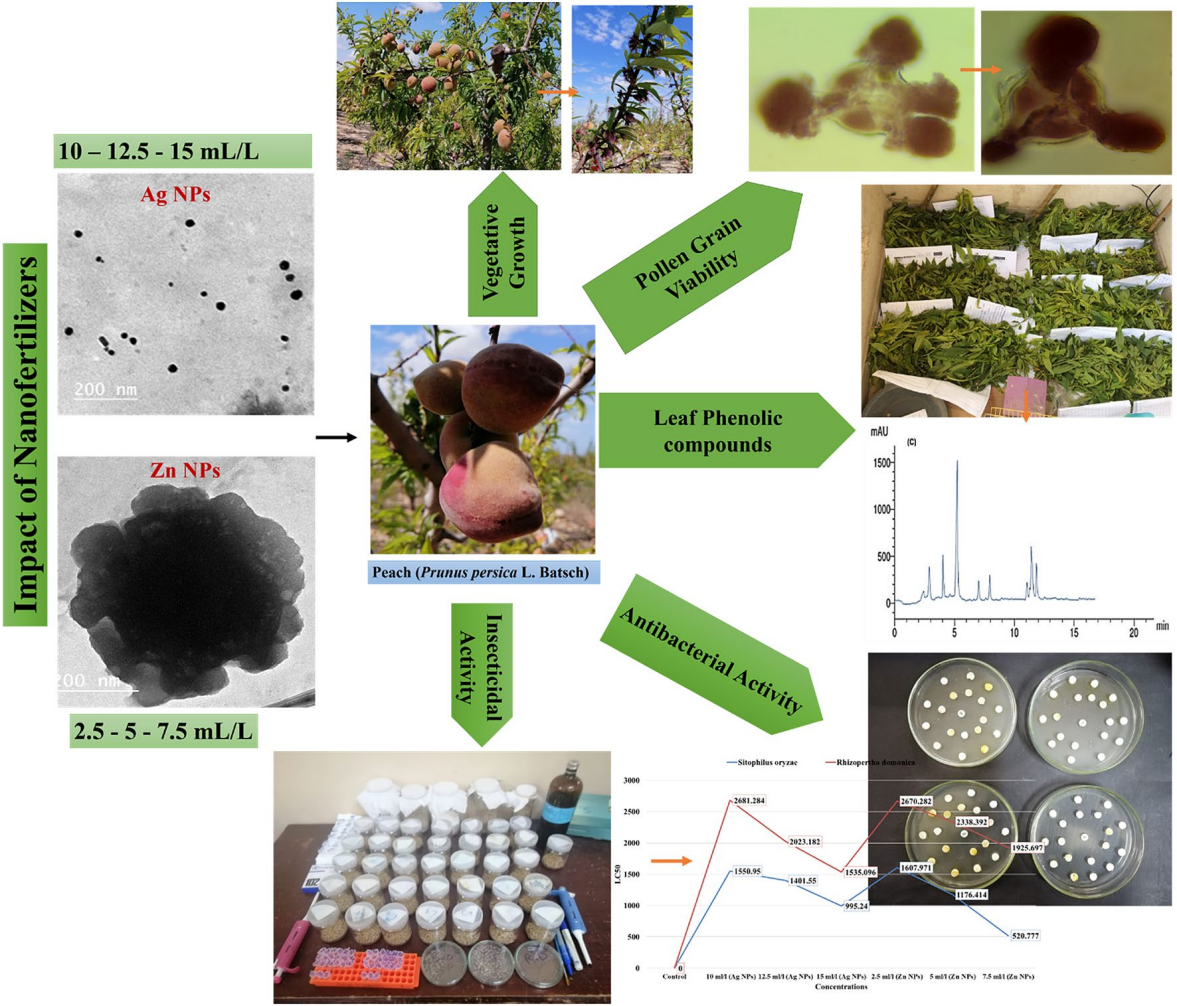
Graphical abstract for the whole experimental steps (Correspondence author, Nader R. Abdelsalam who drawn the graphical abstract).

The current investigation steps and graphical abstract for the whole experimental steps can be summarized in Scheme 1.

### Preparation of nanofertilizers

The commercial product Ag NPs “LINS-MF14” and Zn NPs were obtained from the company "Lotus Middle East Pharma^®^, Egypt". The products were received from the company in a very high concentration (200 ppm), and without any characterization. As per the datasheet provided by the company, serial dilution with deionized water was performed to prepare the following working concentrations (Ag NPs) 10, 12.5, and 15 mL/L and Zn NPs (2.5, 5, and 7.5 mL/L).

#### Characterization of Ag NPs

First, the particle shape and particle distribution were determined using "transmission electron microscope": “TEM; JEOLJEM- 1230; Japan”. The stock concentrated solution of LINS-MF14 (Ag NPs) was centrifuged at 20,000 rpm for 60 min to give Ag NPs in a powder form. The obtained Ag NPs powder was observed using “scanning electron microscopy; SEM: Quanta 400, Oxford, UK” to inspect the morphological characteristics of LINS-MF14 (Ag NPs).

#### Characterization of Zn NPs

The Zn NPs shape was examined by transmission electron nanoscopy (TEM) (JEOL-TEM 100 CX) at the Electron Nanoscopic Unit, Faculty of Science, Alexandria University. Analyses of particles were performed using an H-7500 transmission electron nanoscope (Hitachi, Japan) with an acceleration voltage of 80 kV. TEM was used to examine particles in suspension. The TEM samples were prepared by placing a drop of the suspended particles on carbon-coated copper grids and allowing water to evaporate. The samples on the grids were dry in 4 min. The particle shapes were determined from the TEM nano graphs^76^.

### Experimental design and site

The current experiment was carried out during the year 2020, on eight-years-old "Florida prince" peach trees, planted at 4 × 4 m apart in a sandy clay loam soil under drip irrigation in a private orchard located at El Omid region, Marsa Matruh governorate, Egypt. The physicochemical analysis of experimental soil was carried out according to^77^ as follow: pH (8.17), EC (2.58 dS/m), Na^+^ (15.2), K^+^ (1.6), Ca^2+^ (5.0), Mg2^+^ (4.0), Cl^−^ (14.5), HCO_3_ (5.0), CaCO_3_ (26.7%) and SO_4_ (6.0). Seventy uniform trees were selected at the same vigor as possible for performing this study and were subjected to the same agricultural practices during the two seasons. The trees were sprayed at three times, before flowering, during the full bloom and one month later with the following treatments: water (control), Ag NPs at 10, 12.5 and 15 mL/L and Zn NPs at 2.5, 5 and 7.5 mL/L. The treatments were arranged in a randomized complete block design where each treatment was composed from ten replicates/ten trees.

### Vegetative parameters

Shoot diameter (cm) and average of leaf area (cm^2^) were measured at the end of the growing seasons where, the average leaf area (cm^−2^) was determined using the following equation which adapted by^78,79^:

$$ LA = - 0.5 + \left( {0.23 \times \frac{L}{W}} \right) + \left( {0.67 \times L \times W} \right) $$

where LA is a leaf area, L is leaf length and W is leaf width.

### Yield per tree and fruit quality

Yield was estimated at the harvest time (April 2020), yield was estimated in kg per each tree/replicate and in ton per hectare.

#### Fruit physical characteristics

Thirty fruits samples, at the harvesting time, were chosen randomly from each replicate/tree to determine physical and chemical characteristics, which contain: fruit weight (g), (the weight of each fruit in gram), flesh fruit weight (g) (the wight of fruit without the weight of kernell), fruit length (cm) and fruit diameter (cm). Fruit firmness (Ib/ inch^2^) was measured by a Magness and Taylor pressure tester with 7/18-inch plunger. Fruit size (cm^3^) was measured by weight the volume of replacement water as cm^3^ after dipping fruit in it. Total soluble solids (TSS %) was measured by using a hand refractometer (ATAGO Co. LTD., Tokyo, Japan), from the fresh-cut peach fruit and the result was expressed as percentage (%).

#### Fruit chemical characteristics

Anthocyanin was determined at the stage of coloration (mg/100 g fresh weight peel) according to^80^. Ascorbic acid content of the juice (Vitamin C mg/100 mg juice) was estimated by titration with 2, 6 dichloro phenol-indo-phenol^81^ and calculated as milli-grams per 100 ml of juice. Total and reducing sugars were estimated calorimetrically using Nelson arsenate—molybdate colorimetric method^82^. Non-reducing sugars were calculated by the difference between total sugars and reducing sugars. Percentage of Titratable acidity in fruit juice 100 berries was determined using an AOAC method^83^. TSS/acid ratio was calculated by dividing the value of TSS over the value of titratable acidity.

### Leaf extraction and Insecticidal Activity

Leaf samples collected from the peach trees treated with nanoparticle concentrations were first air-dried at room conditions for 10 days and then ground to powder using a small laboratory mill. About 50 g from each treatment's powdered leaves were extracted by 100 mL acetone by soaking method for one week^45,84^. The extracts were then filtered throughout filter paper (Whatman no.1), and the extracts were concentrated by evaporating the solvent and stored in brown vials for further analysis.

Leaf acetone extracts were determined for their insecticidal activity against some stored-product insects adults of the rice weevil (*Sitophilus oryzea* L.) and the lesser grain borer (*Rhyzopertha dominica*, Fabricius)). The fumigation bioassay method used three concentrations from each Ag NPs (10, 12.5, and 15 mL/L) and Zn NPs (2.5, 5, and 7.5 mL/L).

#### Insect culture

The rice weevil *S. oryzea* (Coleoptera, Curculionidae) and lesser grain borer *R. dominica* (Coleoptera, Bostrichidae) are considered primary storage insects which reared using autoclaved wheat grains in 1-L glass jars covered by fine mesh cloth for ventilation according to the method of^6,85^. Adult insects used in bioassay were about 14 days old. Both culture breeding and experimental procedures were carried under the same laboratory condition (27 ± 1 °C and 65 ± 5% R.H).

#### Fumigation toxicity bioassay

Twenty adults were exposed from each insect *S. oryzea*, *R. domonica* to vapor toxicity by transferring into glass jars (250 ml/L) containing 20 g of sterilized wheat grains. The inner surface of the screw lid of the glass jars was attached with filter papers (9 cm diameter), which applied with different doses of extracts from tree leaves treated with Ag NPs (10, 12.5, and 15 mL/L) and Zn NPs (2.5, 5 and 7.5 mL/L) dissolved in (100 µL) acetone. Before closing, the jars allow the solvent to evaporate for 5 min. Control jars were treated with acetone alone. According to^86,87^, all treatment and control were replicated three times. Mortality percentage (M%) was determined for each concentration, and LC_50_ (lethal concentration 50%) values were calculated according to^88^.

### Bacterial identification and antibacterial activity

#### Bacterial isolation

Pathogens isolation were performed from infected pear and cabbage leaves, guava root galls, and potato tubers that exhibit symptoms and retrieved from the Beheira Governorate, Egypt. The plant materials were thoroughly rinsed, inserted for 30 s in 1% sodium hypochlorite, cleaned in sterile distilled water, and left to dry. Pieces of plant samples were grinded with 0.9% sodium chloride buffer, a loopful streaked on nutrient agar dishes, and incubated at 28 ± 2 for 48 h^89^. The appeared colonies were purified, cultured, and kept at 4 °C for further analysis.

#### Bacterial morphological and molecular identification

The phenotypic characteristics of bacterial isolates were described according to^89^. DNA extraction was done for all purified bacterial isolates, and the template DNA was used in Techne PCR machine (Cambridge, UK) to amplify a 1550 bp fragment of the 16S rDNA gene. The PCR was performed in total volume 25 µl consisting of P0 (5′-GAAGAGTTTGATCCTGGCTCAG-3′), P6 (5′-CTACGGCTACCTTGTTACGA-3′) primers. The partial amplicons were purified, sequenced at Macrogen Inc. (Seoul, South Korea), and further, the sequences were accessioned in the Genbank^90,91^**.**

#### Antimicrobial activity

The antibacterial activity of acetone extract of treated peach leaves with Ag NPs or Zn NPs was tested against all isolates of bacteria obtained in this study compared to control according to the National Committee for Clinical Laboratory Standards^92^.

### HPLC analysis of phenolic compounds in leaf extracts

The phenolic compounds from the acetone extract from each of the treated leaves with nanoparticles were identified using HPLC-Agilent 1100, (Agilent, Santa Clara, CA, USA), which is composed of a quaternary pump and UV/Vis detector. C18 column (125 mm × 4.60 mm, 5 µm particle size). Chromatograms were obtained and analyzed using the Agilent ChemStation. Phenolic compounds were separated by employing a mobile gradient phase of water/acetonitrile/glacial acetic acid (980/20/5, v/v/v, pH 2.68) and acetonitrile/glacial acetic acid (1000/5, v/v) with flow rate at 1 mL/min and detected at 325 nm. All chemical standards (HPLC grade) were purchased from Sigma-Aldrich (St. Louis, MO, USA).

### Assessment of pollen grain fertility

During this study only, mature pollen was used. At the flowering stage, morning from 9 to 11 clock, the anthers of peach were selected from the field after different nano fertilizer treatments and control to study pollen grains fertility^66,70^. To estimate the pollen grain viability, the acetocarmine coloration of the pollen grain was used pollen grains of normal size, which were stained well with acetocarmine were considered fertile. In contrast, those which were stained appeared shrunken, partially filled, and smaller in size than normal were deemed to be sterile^93,94^. Pollen awns, glumes, and lemmas were carefully removed and sampled when lodicules swelled, the stigma fanned out, filaments stretched, and anthers enlarged and turned greenish to bright yellow. Earlier the tip of the anther opened, at least five anthers were transported, and pollen shedding was supported by opening gently with a needle. Nearly 1000 pollen grains were examined and estimated. One drop of aceto-carmine solution was transferred onto the slide, the pollen grains of fresh mature buds were scattered on the slides, the coverslip was placed gently on the slide, and pollen grains' viability was tested.

### Statistical analysis

The gained data were subjected to one-way analysis of variance according to^95,96^. A least significant difference at 0.05% was used to compare between the means of the treatments and measured with CoHort Software (Pacific Grove, CA, USA).

### Compliance with ethical standards

This study is complied with relevant institutional, national, and international guidelines and legislation. “This study does not contain any studies with human participants or animals performed by any of the authors.”

## Results

### Characterization and impact of nanofertilizers

**Figure 1:**
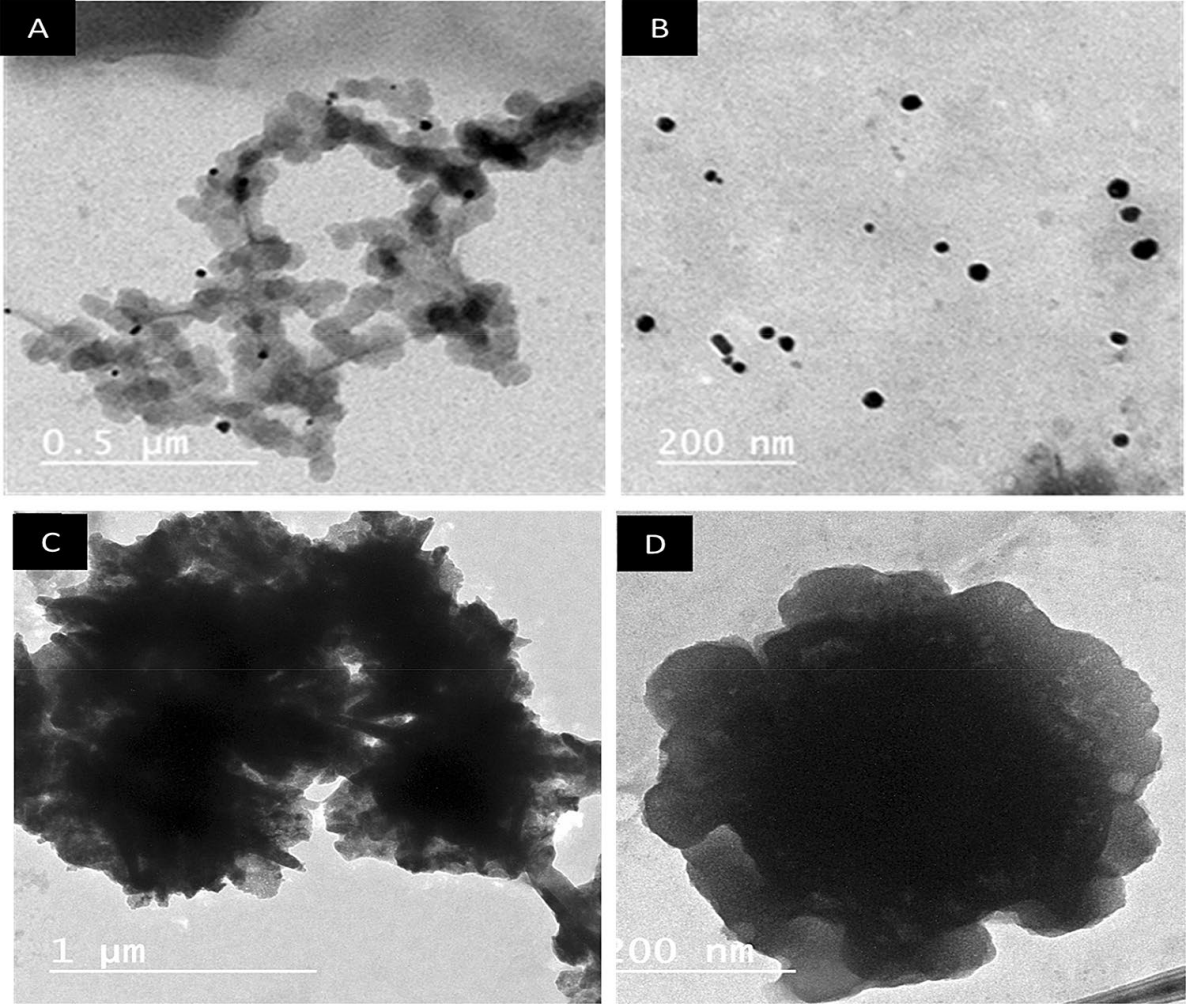
TEM of Ag NPs (**A**,**B**) and Zn NPs (**C**,**D**) at low and high magnifications power.

This work was to evaluate a commercial nano-products called Ag NPs and Zn NPs as nanofertilizers and study their effect on the vegetative growth, pollen grain fertility, yield, and fruit quality of peach. The challenge in this current work was to find out the best concentration of these nanofertilizers that can improve vegetative growth parameters,pollen viability, and yield, fruit physical and chemical characteristics of peach. To characterize the commercial nanoparticles, TEM was used for the detection of the particle shape. It was observed at two different magnifications, as shown in Fig. 1A–D. The analysis was performed by diluting the original sample, Ag NPs, with deionized water followed by ultrasonication for 5 min at room temperature. It was depicted that the particle's shape was small, spherical size (around 40–60 nm). The concentrated available sample as obtained from the company makes it feasible for industrial application in several domains. Below are the commercial fertilizer Zn NPs physical characteristics using TEM, scanning electron nanoscopy SEM. Figure 1C,D shows the commercial fertilizer's particle shape; according to the Zn NPs, the sample has no clear shape with noticeable agglomeration, which shows that the material has very high size particles.

### Vegetative growth parameters, flower percentage and fruit yield

Data in Table 1 cleared that shoots thickness, leaf area, and total chlorophyll were significantly improved by the foliar application of Ag NPs at 10, 12.5 and 15 mL/L and Zn NPs at 2.5, 5 and 7.5 mL/L compared with control. The highest values were obtained by using Ag NPs at 15 mL/L, which was the superior treatment comparing with the rest applied treatments and control. Besides, using 12.5 mL/L Ag-NP and 7.5 mL/L Zn NPs also have a positive effect in improving the same vegetative growth parameters comparing with the other applied treatments and control. Flower %, yield (kg/ tree) and yield (t/h) were remarkably raised by the foliar application of Ag and Zn NPs compared to control. The best increments were obtained by spraying Ag NPs at 12.5 and 15 mL/L and Zn at 7.5 mL/L more than the other applied treatments and control. Besides, the superior treatment was Ag NPs at 15 mL/L ppm, as compared to the rest treatments.

**Table 1:** Influence of spraying Ag and Zn NPs on shoot thickness, leaf area, total chlorophyll, flower percentage, and yield of “Florida prince” peach cultivar during 2020.

Treatments	Shoot thickness (mm)	Leaf area (cm^2^)	Total cholorophyll spad (μ Molm^−2^)	Flower, %	Yield (kg)/tree	Yield (ton)/hectare
Control	0.27^c^	31.88^f^	32.07^d^	36.20^d^	105.25^e^	63.15^e^
Ag NPs 10 mL/L	0.32^b^	37.16^d^	39.93^c^	57.09^b^	140.75^cd^	84.45^cd^
Ag NPs 12.5 mL/L	0.35^ab^	41.54^c^	42.70^a^	57.71^b^	149.50^b^	89.7^b^
Ag NPs 15 mL/L	0.37^a^	48.37^a^	43. 19^a^	70.81^a^	165.00^a^	99.0^a^
Zn NPs 2.5 mL/L	0.31^bc^	34.86^e^	40.90^bc^	54.17^c^	140.25^d^	84.15^d^
Zn NPs 5 mL/L	0.33^ab^	38.72^d^	41.74^ab^	57.63^b^	142.5^cd^	85.5^cd^
Zn NPs 7.5 mL/L	0.35^ab^	44.31^b^	43.01^a^	57.63^b^	146.25^bc^	87.75^bc^
LSD = 0.05	0.05	1.57	1.53	2.84	5.86	3.52

*Means not sharing the same letter(s) within each column, significantly different at 0.05 level of probability.

### Fruit physical characteristics

Data in Table 2 demonstrated that all treatment were greatly increased by spraying Ag NPs 10, 12.5 and 15 mL/L and Zn NPs at 2.5, 5 and 7.5 mL/L comparing with control. The best results were obtained by using 12.5, 15 mL/L Ag NPs, 7.5, and 5 mL/L Zn NPs over control. The superior treatment was spraying Ag NPs at 15 mL/L, which gave the highest values over the other applied treatments. Foliar spraying of Ag NPs at 10, 12.5 and 15 mL/L and Zn NPs at 2.5, 5 and 7.5 mL/L increased the fruit firmness as compared to control but the increment was so slight not enough to be significant and the highest value was obtained by using Ag NPs at 15 mL/L.

**Table 2:** Influence of spraying Ag and Zn NPs on weight, length, diameter, size, flesh weight, and firmness of fruit of “Florida prince” peach cultivar during 2020.

Treatments	Fruit weight (g)	Fruit length (cm)	Fruit diameter (cm)	Fruit size (cm^3^)	Flesh weight (g)	Fruit firmness (Ib/inch^2^)
Control	56.96^d^	3.82^f^	3.62^e^	73.96^e^	51.09^d^	11.3
Ag NPs 10 mL/L	75.73^bc^	4.70^d^	4.73^c^	93.71^d^	68.63^bc^	11.35
Ag NPs 12.5 mL/L	78.06^b^	4.97^b^	4.94^a^	97.06^b^	71.33^b^	11.47
Ag NPs 15 mL/L	84.82^a^	5.28^a^	5.01^a^	106.82^a^	78.02^a^	11.6
Zn NPs 2.5 mL/L	74.88^c^	4.47^e^	4.60^d^	92.88^d^	68.16^c^	11.42
Zn NPs 5 mL/L	76.01^bc^	4.81^c^	4.83^b^	94.01^cd^	69.16^bc^	11.4
Zn NPs 7.5 mL/L	76.71^bc^	5.00^b^	4.75^bc^	96.73^bc^	69.56^bc^	11.47
LSD = 0.05	2.73	0.06	0.09	2.73	2.81	0.58^ns^

*Means not sharing the same letter(s) within each column, significantly different at 0.05 level of probability.

*ns* not significant.

### Fruit chemical characteristics

Data in Table 3 showed that TSS (total soluble solids), total, reduced and non-reduced sugars percentages, TSS/Acidity, vitamin C and anthocyanin content were significantly increased by the foliar spraying of Ag NPs at 15, 12.5 mL/L and Zn NPs at 7.5 mL/L over the rest applied treatments and control. On the opposite side, they reduced statistically the fruit acidity percentage as compared to control. Moreover, the spray of tree with Ag NPs at 10 mL/L and Zn NPs at 5 and 2.5 mL/L also raised clearly also the fruit content from TSS, total, reduced and non-reduced sugars percentages comparing with control. The highest values were obtained by using of Ag NPs at 15 mL/L comparing with the rest treatments.

**Table 3:** Influence of spraying Ag and Zn NPs on TSS, total sugars, reducing sugars and non- reduce sugars, acidity percentages and anthocyanin of fruit of “Florida prince” peach cultivar during 2020 season.

Treatments	TSS (%)	Total sugars (%)	Reduced sugars (%)	Non-reduced sugars (%)	TSS/acidity	Acidity (%)	V.C. Mg/100 ml	Antho-cyanine (mg/100 g)
Control	8.29^f^	6.08^f^	2.26^f^	3.81^e^	12.50^b^	0.67^a^	5.08^d^	0.30^d^
Ag NPs 10 mL/L	8.97^d^	7.06^d^	2.84^c^	4.23^d^	14.71^b^	0.61a^bc^	5.65^c^	0.36^c^
Ag NPs 12.5 mL/L	10.04^b^	8.14^b^	3.20^a^	4.94^b^	17.96^a^	0.56^bc^	7.14^a^	0.44^b^
Ag NPs 15 mL/L	10.51^a^	8.59^a^	3.25^a^	5.34^a^	19.58^a^	0.54^c^	7.41^a^	0.57^a^
Zn NPs 2.5 mL/L	8.70^e^	6.67^e^	2.49^e^	4.18^d^	13.58^b^	0.64^ab^	5.35^cd^	0.30^d^
Zn NPs 5 mL/L	9.06^d^	6.96^d^	2.70^d^	4.26^d^	14.41^b^	0.63^ab^	5.52^c^	0.33^cd^
Zn NPs 7.5 mL/L	9.62^c^	7.73^c^	3.08^b^	4.64^c^	18.02^a^	0.54^c^	6.33^b^	0.57^a^
LSD = 0.05	0.13	0.12	0.09	0.12	2.44	0.09	0.43	0.04

*Means not sharing the same letter(s) within each column, significantly different at 0.05 level of probability.

### The impact of nanofertilizers (leaf extracts of Peach) against stored product insects

**Figure 2:**
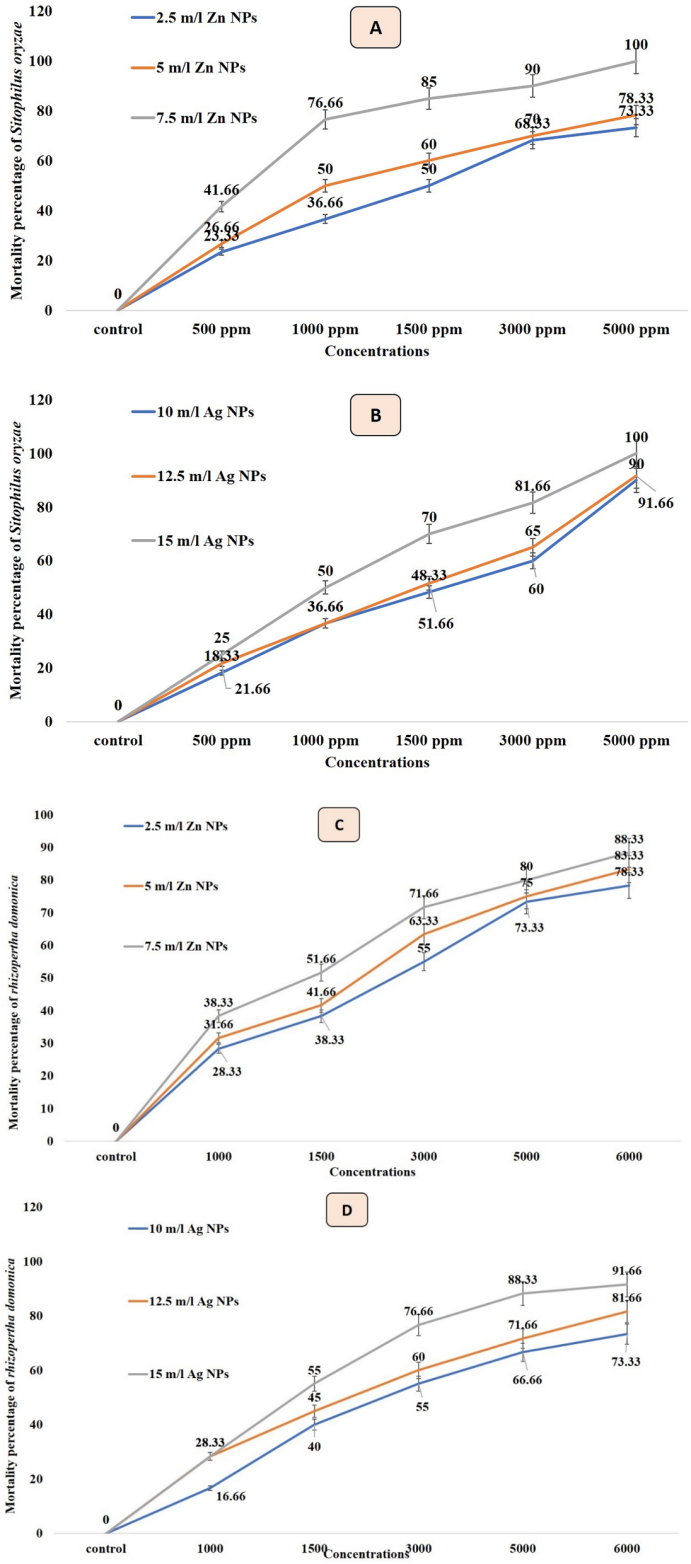
(**A**) Effect of acetone extracts from leaves of trees treated Zn NPs (a) and Ag NPs (b) on mortality percentage of *Sitophilus oryzae*. (**B**) Effect of acetone extracts from leaves of trees treated Zn NPs (c) and Ag NPs (d) on mortality percentage of *Rhizopertha domonica.*

**Figure 3:**
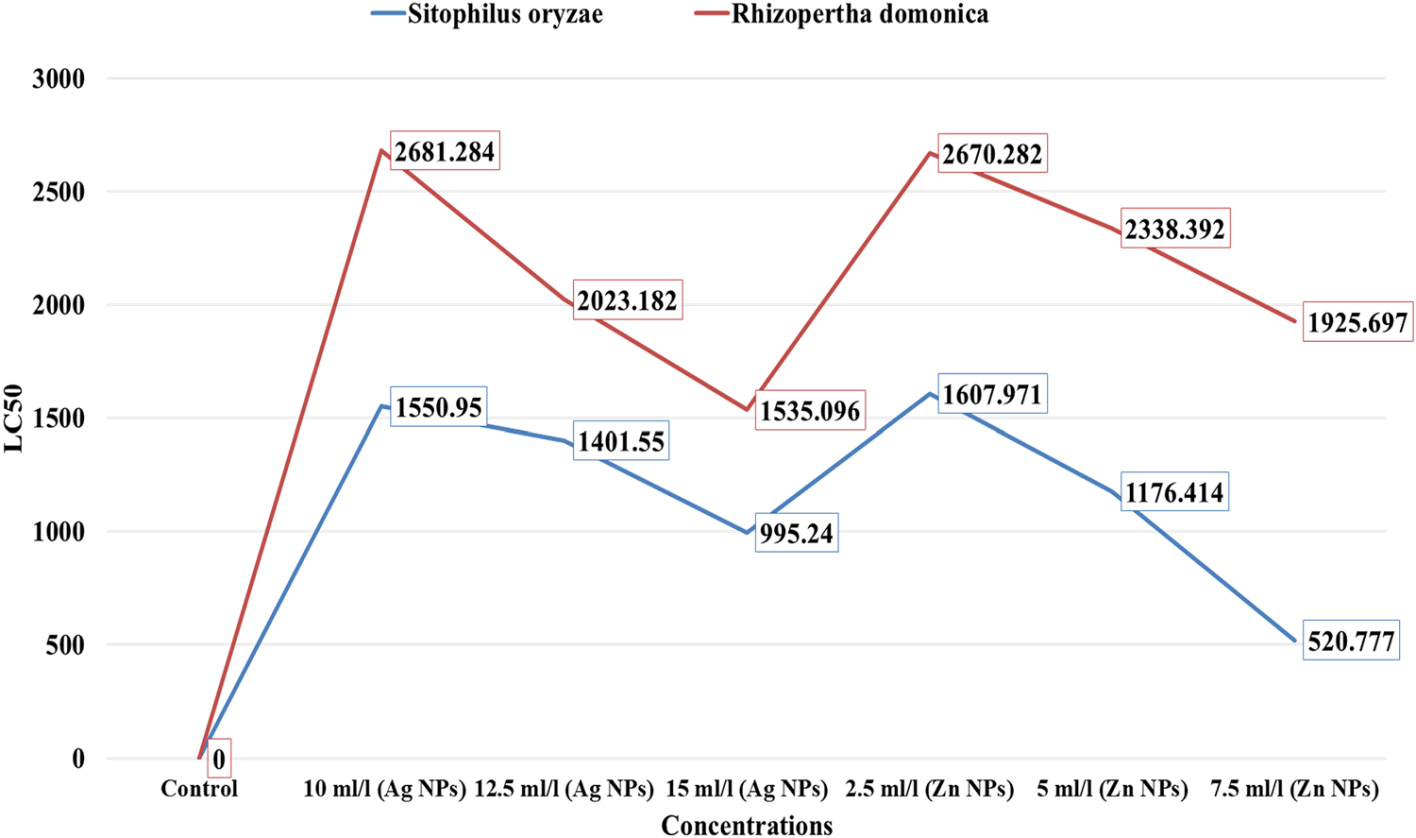
Insecticidal activity of acetone leaf extracts from peach trees treated with different concentrations of Zn NPs and Ag NPs against *Sitophilus oryzae* and *Rhizopertha domonica.*

Data in Fig. 2A,B indicate that the leaf acetone extract with an increase of nanomaterials concentration increases the mortality percentage for both insects. The results revealed high efficiency of an acetone leaf extract from treated trees with Ag NPs compared with Zn NPs. On the other hand, the rice weevils *Sitophilus oryzae* was more susceptible than *Rhizopertha domonica* which showed more resistance to these extracts. Extracts from leaves treated with 7.5 and 15 mL/L of Zn and Ag NPs showed 100% mortality percentage at 5000 ppm for the rice weevils *S oryzae* and 88.33 and 91.66% mortality percentage at 6000 ppm for *R. domonica..* Table 4 and Fig. 3 present the different values of LC_50_ and LC_90_ of acetone extracts from leaves sprayed with Ag and Zn NPs under the different concentrations; for instance, the extract from leaves treated with Ag NPs showed LC_50_ against the rice weevils *S. oryzae* ranged from 955.24 (range 645.03–1535.57 ppm under 15 mL/L) to 1550.95 (range 1001.54–2401.75 ppm under 10 mL/L). While LC_90_ ranged from 4153.16 (range 2691.75–6407.99 ppm under 15 mL/L) to 7034.61 (range 4542.66–10,893.54 ppm under 10 mL/L). The lowest LC_50 and 90_ values from extracts obtained from leaves treated with Zn NPs were recorded under the 7.5 mL/L as 520.77 (range 312.137–868.875 ppm) and 2501.219 (range 1499.152–4173.09 ppm), respectively, against the rice weevils *S. oryzae*.

**Table 4:** Insecticidal activity of leaf acetone extracts from trees treated with different concentrations of Ag and Zn NPs against *Sitophilus oryzae* and *Rhizopertha domonica.*

Leaf Acetone extracts from treated trees with	LC_50_	Lower limit	Upper limit	LC_90_	Lower limit	Upper limit	Slope ± SE
***Sitophilus oryzae***							
Control	0	0	0	0	0	0	0
Ag NP 10 mL/L	1550.95	1001.54	2401.75	7034.61	4542.66	10,893.54	1.992 ± 0.097
Ag NP 12.5 mL/L	1401.55	909.78	2159.14	6216.50	4035.29	9576.72	2.026 ± 0.096
Ag NP 15 mL/L	995.24	645.03	1535.57	4153.16	2691.75	6407.99	2.073 ± 0.096
Zn NP 2.5 mL/L	1607.971	892.238	2897.848	12,905.332	7160.970	23,257.688	1.419 ± 0.131
Zn NP 5 mL/L	1176.414	632.697	2187.383	10,548.166	5672.995	19,612.886	1.350 ± 0.137
Zn NP 7.5 mL/L	520.777	312.137	868.875	2501.219	1499.152	4173.090	1.909 ± 0.113
***Rhizopertha domonica***							
Control	0	0	0	0	0	0	0
Ag NP 10 mL/L	2681.284	1694.526	4242.653	13,544.510	8559.899	21,431.766	1.840 ± 0.102
Ag NP 12.5 mL/L	2023.182	1236.030	3311.623	11,390.064	6958.574	18,643.699	1.714 ± 0.109
Ag NP 15 mL/L	1535.096	1045.373	2254.238	5298.789	3608.380	7781.100	2.386 ± 0.085
Zn NP 2.5 mL/L	2670.282	1819.723	3918.402	9846.303	6709.982	14,448.575	2.261 ± 0.085
Zn NP 5 mL/L	2338.392	1599.363	3418.908	8464.648	5789.468	12,375.966	2.296 ± 0.084
Zn NP 7.5 mL/L	1925.697	1315.068	2819.859	6883.453	4700.746	10,079.661	2.323 ± 0.085

Regarding the *R. domonica*, which was treated with different Ag NPs and Zn NPs treatments, the results in Table 4 and Fig. 3 showed the different LC_50 & 90_ under different concentrations. As observed before, this insect showed higher resistance to both nanomaterilas compred with *S. oryzae* and recorded a lower mortality percentage. For example, the different concentrations of Ag NPs against lesser grain borer *R. domonica* showed LC_50_ ranged from 1535.096 (range 1045.373–2254.238 ppm under 15 mL/L) to 2681.284 (range 1694.526–4242.653 ppm under 10 mL/L). While LC_90_ ranged from 5298.789 (range 3608.380–7781.100 ppm under 15 mL/L) to 13,544.510 (range 8559.899–21,431.766 ppm under 10 mL/L). The lowest LC_50 and 90_ of Zn NPs were recorded under the 7.5 mL/L as 1925.697 (range 1315.068–2819.859 ppm) and 6883.453 (range 4700.746–10,079.661 ppm), respectively against lesser grain borer *R. domonica* (Table 4 and Fig. 3).

### The impact of nanofertilizers (leaves extract of peach) as antibacterial activity

Four bacterial isolates were retrieved from pear, guava, cabbage, and potato plants. The phenotypic features and the 16S rDNA gene sequences identified the isolates as *Erwinia amylovora*, *Agrobacterium tumefaciens*, *Dickeya solani*, and *Serratia pylumthica* (Table 5). The acetone extract's antibacterial activity from the peach leaves treated with Ag NPs, or Zn NPs is shown in Table 6. In general, the extract concentrations did not exhibit antibacterial activity against the growth of all bacterial strains used in this study except the Ag NPs treated leaves extract at conc. 3000 µg/mL gave inhibition zone (IZ) ranged from 8.67, 7.33, 6 and 6 mm for *E. amylovora, A. tumefaciens*, *D. solani,* and *S. pylumthica*, respectively compared to control where the IZ reached 8.33 mm in the antibiotic treatment.

**Table 5:** Morphological, physiological, biochemical characteristics, and accession numbers of bacterial isolates retrieved from infected plants.

Characteristic	Bacterial isolate			
	*Erwinia amylovora*	*Agrobacterium tumefaciens*	*Dickeya solani*	*Serratia plymuthica*
Accession number	MK720289	MK720286	MK720280	MK720276
Shape (rods)	+	+	+	+
Motility	+	+	+	+
3-Ketolactose production	+	−	+	+
Growth at 37 °C	−	−	+	+
Oxidase reaction	−	+	−	−
Indole production	−	−	+	−
Growth in 5% NaCl	−	−	−	+
Citrate utilization	−	+	+	−
Malonate utilization	−	−	+	−
Alkali from tartaric acid	nd	+	+	−
Glucose	a	−	+	+
α-methyl glucoside	−	−	a	a
Maltose	−	−	−	a
Sucrose	a	a	a	a
Dulcitol	a	a	a	a

+  = More than 80% of isolates gave a positive reaction. −  = Less than 20% of isolates gave a negative reaction.

*A* acid, *nd* not determined.

**Table 6:** Treatments and bacterial isolates inhibition zone diameter (mm) calculations.

Treatments	Concentrations, µg/ml	Bacterial isolates inhibition zone diameter (mm)			
		*Erwinia amylovora*	*Agrobacterium tumefaciens*	*Dickeya solani*	*Serratia plymuthica*
DMSO	–	–*	–	–	–
Amoxicillin antibiotic	25	7.33	7	8.33	6.33
Leaves extract treated with Ag NPs	3000	8.67	7.33	6	6
	2000	–	–	–	–
	1000	–	–	–	–
Leaves extract treated Zn NPs	3000	–	–	–	–
	2000	–	–	–	–
	1000	–	–	–	–
Leaves non treated (control)	3000	–	–	–	–
	2000	–	–	–	–
	1000	–	–	–	–

*–, mean there no effect.

### Phenolic compounds analyzed by HPLC

**Figure 4:**
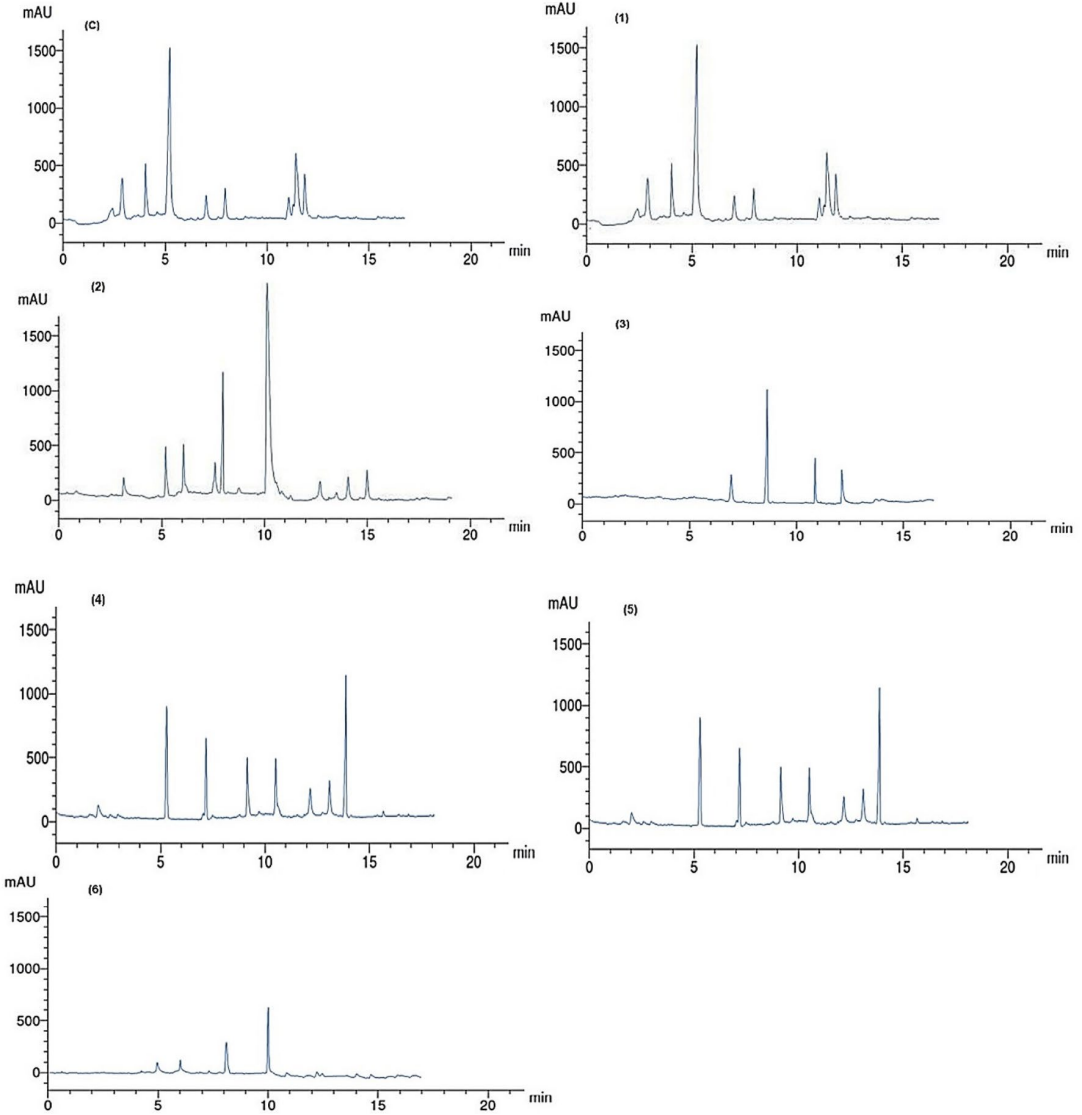
HPLC chromatograms of phenolic compounds identified in acetone extract from leaves of peach trees treated with (**1**) Ag NP 10 mL/L, (**2**) Ag NP 12.5 mL/L, (**3**) Ag NP 15 mL/L, (**4**) Zn NP 2.5 mL/L, (**5**) Zn NP 5 mL/L, and (**6**) Zn NP 7.5 mL/L compared to untreated trees (C).

Table 7 and Fig. 4 present the changes in phenolic compounds of acetone leaf extracts from peach trees as affected by different treatment concentrations of nanoparticles. Catechol found in 4.22 mg/g in extract from leaves sprayed with Ag NPs 10 mL/L and control (without foliar application) and decreased to 1.22 mg/g in leaves treated with Ag NPs 12.5 mL/L, and then it was not detected in other treatments. Caffeic acid was found in 6.15 mg/g in both leaves from control and treated with Ag NPs 10 mL/L and not detected in other treatments.

**Table 7:** HPLC analysis of phenolic compounds in leaves of peach (*Prunus persica* L. Batsch).

RT (min)	Compound	Phenolic compounds (mg/g) in acetone extract of peach leaves						
		Control	Ag NPs 10 mL/L	Ag NPs 12.5 mL/L	Ag NPs 15 mL/L	Zn NPs 2.5 mL/L	Zn NPs 5 mL/L	Zn NPs 7.5 mL/L
2.98	Catechol	4.22	4.22	1.22	ND	ND	ND	ND
4.0	Caffeic acid	6.15	6.15	ND	ND	ND	ND	ND
5.0	Ferulic acid	17.09	17.09	5.10	ND	12.09	12.09	0.07
6.0	p-Coumaric acid	ND	ND	6.06	ND	ND	ND	0.03
7.0	Gallic acid	2.33	2.78	2.78	1.22	11.14	11.14	ND
8.0	Chlorogenic acid	3.14	5.14	8.14	ND	ND	ND	5.22
8.9	*p*-Hydroxybenzoic acid	ND	ND	ND	10.66	8.55	8.55	ND
10.0	Cinnamic acid	ND	ND	14.17	ND	7.43	7.43	9.14
11.0	Salicylic acid	1.05	6.13	ND	7.51	ND	ND	ND
11.5	Epicatechin	7.11	9.03	ND	ND	ND	ND	ND
12.0	Ellagic acid	6.88	7.63	1.05	6.24	1.22	1.22	ND
13.0	Pyrogallol	ND	ND	ND	ND	1.36	1.36	ND
14.0	Protocatechuic acid	ND	ND	3.10	ND	16.09	14.36	ND
15.0	Tyrosol	ND	ND	4.19	ND	ND	ND	ND

*ND* not detected.

The high amount of ferulic acid detected in extract from leaves treated with Ag NPs 10 mL/L and control (17.09 mg/g) and in Zn NPs at 2.5 and 5 mL/L (12.09 mg/g). High amounts of p-Coumaric acid (6.06 mg/g) in extract from leaves treated with Ag NP 12.5 mL/L, gallic acid (11.14 mg/g) from leaves treated with Zn NPs at 2.5 and 5 mL/L, chlorogenic acid (8.14 mg/g) when leaves treated with Ag NPs 12.5 mL/L, p-hydroxybenzoic acid with 10.66 mg/g (leaves treated with Ag NP 15 mL/L) and 8.55 mg/g (leaves treated with Zn NPs at 2.5 and 5 mL/L), cinnamic acid at 14.17 mg/g as leaves sprayed with Ag NP 12.5 mL/L, salicylic acid at 6.13 and 7.51 mg/g when application of Ag NPs at 10 and 15 mL/L, was used, epicatechin at 7.11 and 9.03 mg/g as leaves in control and sprayed with Ag NPs10 mL/L, respectively, ellagic acid was found at high amounts of 6.88 mg/g (control trees), 7.63 mg/g (trees sprayed with Ag NPs 10 mL/L) and 6.24 mg/g (trees sprayed with Ag NPs 15 mL/L), pyrogallol (1.36 mg/g) and as trees treated with Zn NPs at 2.5 and 5 mL/L, protocatechuic acid at 16.09 and 14.36 mg/g as trees treated with Zn NP at 2.5 and 5 mL/L, respectively, and finally tyrosol was only found in leaves treated with 4.19 mg/g Ag NP 12.5 mL/L.

### The impact of nanofertilizers on Pollen grains viability

**Figure 5:**
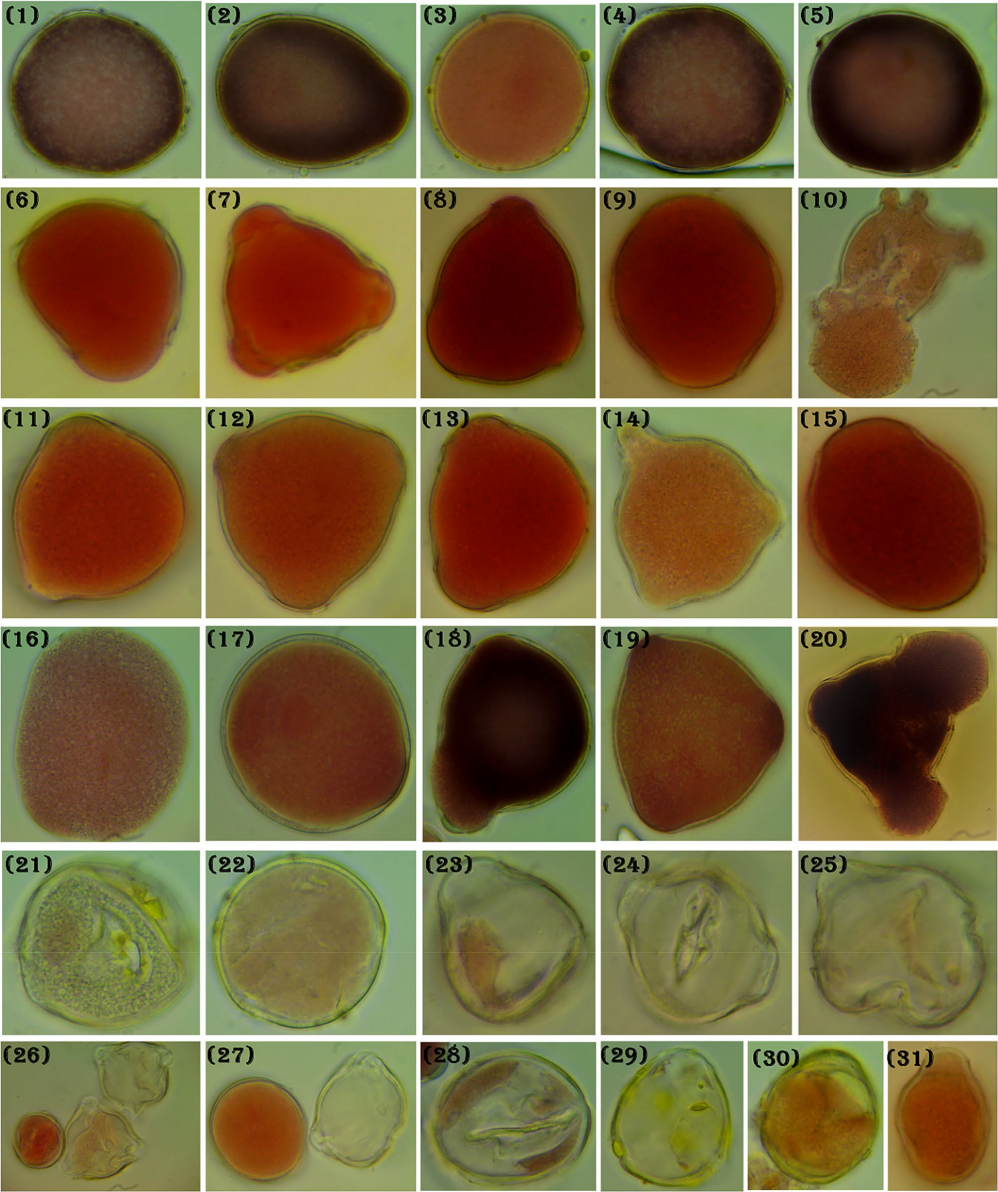
Showing the pollen grains viability and aberration of Zn NP as follow: (**1**–**5**) control, (**6**–**10**) 2.5 mL/L, (**11**–**15**) 5 mL/L, (**16**–**12**) 7.5 mL/L and (**21**:**31**) different types of aberrations on Florida prince” peach cultivar during 2020 season (100 ×).

**Figure 6:**
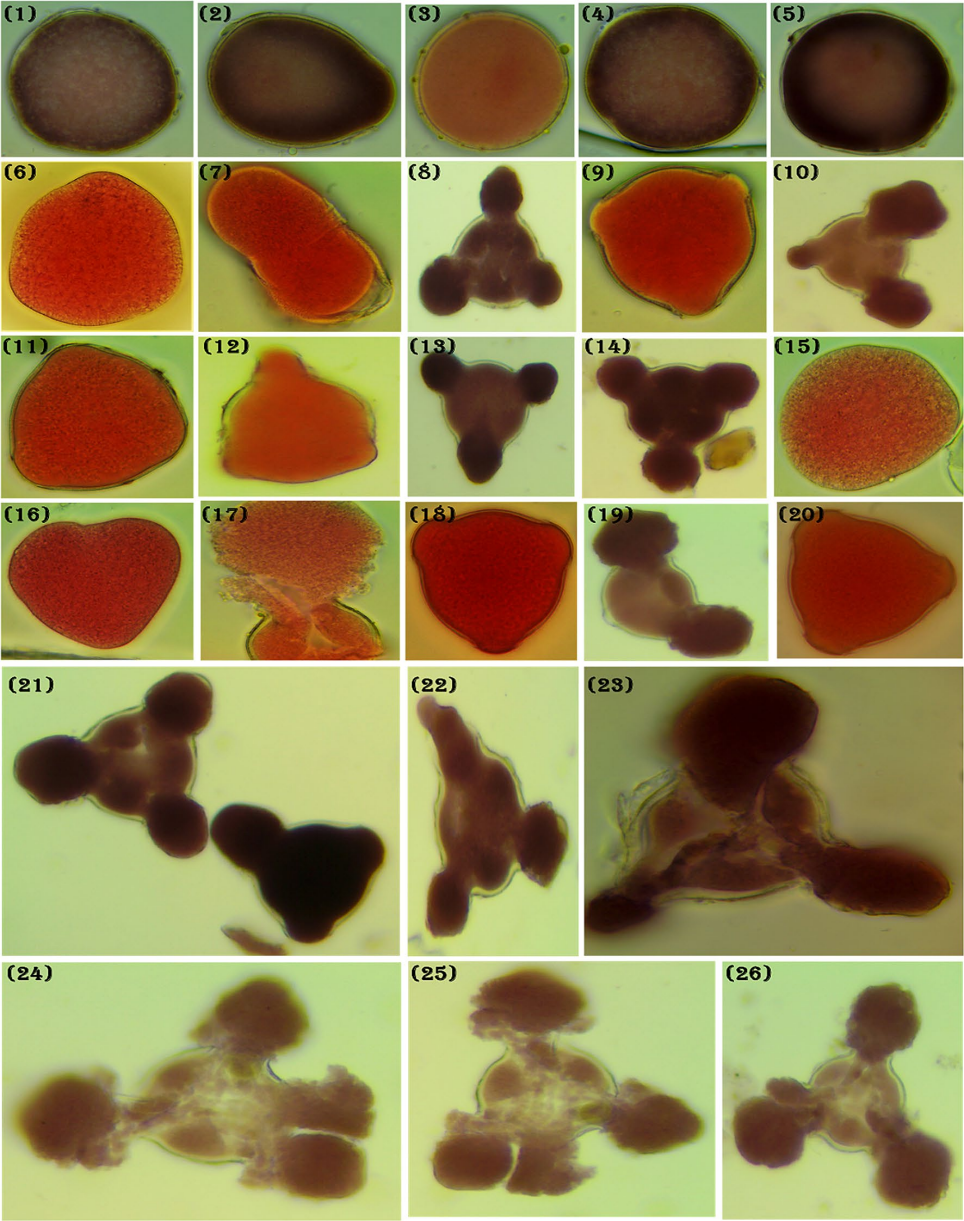
Showing the pollen grains viability and aberration of Ag- NPs (LINS-MF14) as follow: (**1**–**5**) control, (**6**–**10**) 10 mL/L, (**11**–**15**) 12.5 mL/L, (**16**–**21**) 15 mL/L and (**21**:**26**) showing the high pollen fertility and increase of spore numbers on Florida prince” peach cultivar during 2020 season (100 ×).

The results indicated that the two commercial nanofertilizers (Zn NPs and Ag NPs) had significant effect on pollen viability, but unfortunately, with Zn NPs, the results in Fig. 5 (No. 21–31) detected different types of pollen aberrations such as stickiness in content, ultrastructural changes in the exine and interior walls of pollen grains, increase in ultrastructural changes, partially or fully degenerated content and shrunken in pollen content with big gap in capacity. On the other hand, the commercial Ag NPs showed high increase in pollen viability without any aberrations was observed. The commercial Ag NP highly increased the spores (Fig. 6, No. 21–26) the pollen size is increased and the spores also increased and separated in different localities searching about the egg for pollination and fertilization, this case is very important in plant breeding and improving, in addition fruit maturity and size.

Data in Fig. 5 (No. 1–31) showing the effect of Zn NPs on pollen structure and staining capacity, from No. 1–5 showing control group with normal size, but still in round form without swelling like other groups; from No. 6–10 showed the effect of using 2.5 mL/L Zn NPs with different three swelling as resulted of high pollen viability; from No. 11–15 recorded for the second treatment (5 mL/L) and from 16 to 20 showed the different forms of pollen under 7.5 mL/L of Zn NP with an increase in size and spores numbers with different swelling. Results in Fig. 5, No. 21:31 showed the different types of aberrations on Florida prince” peach cultivar during 2020 season as affected by Zn NPs. The observation in Fig. 6 was at the same trend in Fig. 5 but without any pollen aberrations. Besides, there were an observed increase in cell size and the length and numbers of swelling due to the great effect of the commercial product Ag NPs.

## Discussion

The obtained results clearly demonstrated the positive effect of the foliar spraying of Ag and Zn NPs in improving the vegetative growth parameters, yield and fruit quality of peach cv. Florida prince. These results are consistent with the findings of many authors, they reported that the usage of Ag NPs in proper doses plays a crucial role in raising the efficacy of water and fertilizers utilization in soybean^97^. The application of Ag NPs on maize plants at 10–50 μl/l raised the rate of chlorophyll while, high condensations gave inverse effect^98^. Moreover, the application of Ag NP increased growth performance of borage^99^. Also^100^, reported that the application of Ag NPs on *Bacopa monnieri* affected positively on seeds germination, while it minimized the content of total phenol^101^. Furthermore, it was stated that Ag NPs have a crucial role in improving the efficacy of photosynthesis quantity and the content of chlorophyll^102^. Ag NPs increased the growth in *Arabidopsis thaliana*^103^ and in *Eruca sativa*. Furthermore^104^, found that the usage of Ag NP on cucumber at different levels raised the yield and quality of fruit. Ag NPs stimulate the operation of photosynthesis in maize^105^. Ag NP improved the stem length of fodder beet^106^. Besides^107^, reported that using Ag NPs at 100 ppm enhanced significantly the yield of onion comparing with control^108^ found that spraying fenugreek plant with Ag NPs at 20, 40 and 60 mg/l increased the yield components, because they improved the growth parameters and the most effective treatment was 40 mg/l compared with untreated plants.

Zinc is an important nutrient for plants, has various physiological roles in higher plants and is involved in the metabolism of proteins, carbohydrates, nucleic acids and lipids, photosynthesis, and biosynthesis of auxin^109,110^. The foliar application of Zn on olive cultivar, greatly raised the fruit quality and minimized the percentage of fruit drop^111^. Moreover, spraying Zn improved the yield and fruit quality in orange^112,113^, grape fruit^114^, and mandarin^115,116^. Also^117^, found that zinc plays a crucial role in enhancing the leaf area because it raises the metabolic operations and prolongation of the plant cell. In addition, zinc is important in tryptophan synthesis, and it has a crucial role in auxin producing which is fundamental hormone of growth^118,119,120^ stated that Zn is very important for photosynthesis operation where it is involved in carbonic anhydrase enzymes synthesis, which is responsible for transferring CO_2_^35^. It was reported by^121^ zinc is necessary to photosynthesis, tryptophan amino acid synthesis and cell division. Zn NPs has raised the seed germination, seedling vigour, leaf chlorophyll content, stem, and root growth in peanut^40^. Besides^122^, found that zinc is important for the enzymes, which are involved in the metabolism of nitrogen, transduction of energy and synthesis of protein, so its deficiency delays the growth and yield of plants. Also^123^, reported that Zn NP at 0.5 and 1 g/L significantly increased growth, fruit weight and yield per tree of mango since these compounds can easily penetrate plant tissues through stomata.

Phenolic and flavonoid compounds neochlorogenic, chlorogenic acids, catechin, epicatechin, gallic, caffeic, syringic, ferulic, coumaric acids, quercetin-3-rutinoside, quercetin-3-galactoside, cyanidin-3-glucoside, and cyanidin-3-galactoside were characterized from the leaves of *Prunus persica*^72^. Phenolic acids p-coumaric, caffeic, ferulic, chlorogenic, gallic, protocatechuic, 3-O-caffeoylquinic, and 5-O-coumaroylquinic, and 3-O-caffeoylquinic acid methyl ester, chlorogenic acid methyl ester, and 3-O-caffeoyl-5-O-coumaroylquinic acid (3-O-coumaroyl-5-O-caffeoylquinic acid), were identified in leaf extract from *Prunus cerasifera* Ldb^124^. Ferulic acid was isolated from stem extract of peach (*Prunus persica* (L.) Batsch)^65,125^.

The present results indicated that Ag and Zn NPs were effective against the *S. oryzae* and *R. domonica* as the main stored products insects, which attained 100% mortality percentage. Current results agree with^6,126,127^, they reported the influence of nanomaterials as an alternative pesticide anti stored grains insects. The present data indicated that nanomaterials could improve to produce new insecticides and these results agreed with^128^**,** who reported the same finding. The use of nanomaterials as alternative pesticides represents a new approach to combat pests, which have become resistant to common chemical pesticides. Current nanomaterials get absorbed into the cuticular lipids by physio sorption and thus affect the insect’s death.

The antibacterial results of peach leaf extracts were opposite to the results achieved by few authors. The normal peach leaves were found to be bactericidal or fungicidal in a study by^129^**,** who found that supercritical carbon dioxide extracted *Prunus persica* leaves had antimicrobial activity against *Escherichia coli, Staphylococcus aureus, Staphylococcus epidermidis, Enterococcus faecalis, Enterococcus faecium,* and *Candida albicans*. In our study, Zn NPs were not effective against all bacterial isolates used. The control treatment extract results did not affect the bacterial isolates' growth, which does not agree with^130^**,** who noticed a broad antibacterial effect of peach’s (*Persica vulgaris* Miller) leaves methanol extract on several gram-positive or negative bacteria. The Ag NPs treatment results also had a moderate antimicrobial impact on *E. amylovora, A. tumefaciens*, *D. solani,* and *S. pylumthica* that in line with the same authors^129,130^**.**

Currently an increase in usage of nanofertilizers worldwide due to their efficiency and decrease the use of chemical fertilization to save human health and the environment, so during this current study, we tested the efficiency of two commercial nanofertilizers on some morphological, yield, and pollen characteristics. Pollen grains viability refers to the pollen's ability to perform its function of delivering male gametes to the embryo sac. The pollen grain's functional property of their release from the anther varies significantly from species to species, and their quality is measured based on the pollen viability. The viable pollen is vital for species distribution, fitness, and survival of the following plant generation. Besides, it is critical for directed plant breeding and crop improvement. The results suggested that using nanofertilization caused successful pollination, and the gametophytes are essential processes for fruit production and improvements. Also, pollen's viability assessment is an important tool to assess the different biological status and pollen capacity to generate tubes, penetrate the stigma, and elongate inside the style until two male gametes are released within the female gametophyte. The current results are agreeing with this data, which reported that the pollen morphology had become a vital descriptor^131^, as the pollen has its own unique set of characteristics such as size, exine structure, and pore size or number^132^. The present investigation considers a useful tool to screen the effect of nanofertilizers on pollen grain viability test because the knowledge of the viability and capacity of pollen grains is crucial for the reproductive biology and genetic breeding of plants, and this finding agreeing with^133^. Current results in line with^134^ reported that studies of pollen germination and pollen tube growth are essential for understanding fertilization and seed formation in flowering plants and are very useful for explaining any lack of plant fertility. Our results indicated a high effect of nanofertilizers on pollen grains, and these results agree with^135,136^**.** Also^137^, detected many factors that could affect pollen germination such as botanic species, cultivar, plant nutritional state, culture medium, temperature, pollen sampling time, photoperiod, sampling method, application of fertilizers or pesticides to plants, and pollen storage conditions. On the contrary, some studies detected the side effect of using nanomaterials, such as^138–142^. On the other hand^6,79,143–147^, revealed a positive effect of the application of nanofertilizers on plant growth.

## Conclusion

Foliar spraying of Ag NPs at 15 mL/L was the best treatment, which gave the best results in improving shoot diameter, leaf area, total chlorophyll, flower percentage, yield, and fruit physical and chemical characteristics, followed by 12.5 mL/L and Zn NPs at 7.5 mL/L as compared to the rest applied treatments and control. The results indicated that with the increase of nanomaterials concentration, there is an increase in mortality percentage for the rice weevils *S. oryzae* and *R. domonica*. Also, there is a high efficiency of Ag NPs compared with Zn NPs; on the other hand, *S. oryzae* was more susceptible than *R. domonica* and showed more resistance to these nanomaterials. In our study, the peach leaves treated with Ag NPs acetone extract had a moderate inhibitory effect on *E. amylovora, A. tumefaciens*, *D. solani,* and *S. pylumthica*, whereas Zn NPs did not have an antimicrobial impact at all. The results also indicated that the two commercial nanofertilizers caused a highly significant effect on pollen viability, unfortunately, Zn NPs detected different types of pollen aberrations.

## Data Availability

The data used to support the findings of this study are included within the article.
